# 

**Published:** 1985-07

**Authors:** 


					CHIRURGICAL
JULY 1985

				

